# Sex- and Region-Dependent Differences in Sharp Wave–Ripples Along the Long Axis of the Hippocampus

**DOI:** 10.3390/cells15121109

**Published:** 2026-06-19

**Authors:** Athina Miliou, Giota Tsotsokou, Michaela Tsouka, Costas Papatheodoropoulos

**Affiliations:** 1 Laboratory of Physiology-Neurophysiology, Department of Medicine, University of Patras, 26504 Rio, Greece; athina.miliou@ac.upatras.gr (A.M.); ptsotsokou@ac.upatras.gr (G.T.); mihaela.tsouka.42@gmail.com (M.T.); 2 Laboratory of Experimental Animals, School of Health Sciences, University of Patras, 26504 Rio, Greece

**Keywords:** sharp wave, ripple, multiunit activity, network dynamics, dorsoventral hippocampus, sex, septotemporal, rat

## Abstract

**Highlights:**

**What are the main findings?**

- Sharp wave–ripples (SWRs) exhibit robust dorsoventral differences along the hippocampus.
- Sex effects on SWRs are selective and do not affect occurrence rate or amplitude.
- SWR-associated neuronal activity shows a significant sex effect, with higher values in males.
- Ripple power is influenced by both sex and hippocampal region.

**What are the implications of the main findings?**

- Selective sex-related effects on SWR-associated neuronal activity and oscillatory properties suggest differences in hippocampal network dynamics between males and females.
- These findings provide a framework for future studies investigating how sex-dependent hippocampal activity relates to cognition, stress processing, and affective function.

**Abstract:**

Sharp wave–ripples (SWRs) are transient hippocampal population events that coordinate neuronal ensemble activity and play a central role in memory consolidation and affective processing. Although SWRs exhibit marked functional specialization along the dorsoventral axis of the hippocampus, and several cellular mechanisms underlying SWRs are sex-sensitive, systematic comparisons of SWR properties between females and males are lacking. Here, we examined sex- and region-dependent differences in SWRs and associated multiunit activity (MUA) in acute hippocampal slices from adult female and male rats. Spontaneous SWRs were recorded from the CA1 stratum pyramidale of the dorsal and ventral hippocampus, and SWR occurrence rate, amplitude, ripple oscillation properties, and SWR-locked neuronal firing were quantified. Linear mixed-effects analysis revealed robust region-dependent differences across multiple SWR parameters. In contrast, sex effects were selective. SWR occurrence rate and amplitude did not differ significantly between females and males, whereas SWR-associated MUA showed a significant main effect of sex, with higher values in males. Ripple power was also influenced by sex, with higher values in females, together with a significant effect of region, suggesting differences in oscillatory structure. Baseline MUA did not differ between sexes, indicating that sex-related effects are specific to the SWR state. These findings suggest that sex does not substantially alter the generation of SWRs per se but influences neuronal recruitment and oscillatory properties during these events. Our results reveal previously underappreciated dimensions of hippocampal network organization and provide a descriptive framework for future studies investigating how sex-dependent circuit properties may shape hippocampal contributions to cognition and affective regulation. They further highlight the importance of incorporating sex as a fundamental biological variable in studies of hippocampal network dynamics.

## 1. Introduction

Sharp wave–ripples (SWRs) are transient, population events generated by the coordinated activity of hippocampal neuronal ensembles, and they consist of a large-amplitude sharp wave originating primarily in CA3 recurrent circuitry and a superimposed ripple oscillation (~120–200 Hz) in CA1 [1]. Their excitatory output influences widespread cortical and subcortical targets and is thought to support key operations of episodic memory, systems consolidation, planning, and decision-making, by enabling the reactivation of experience-dependent neuronal assemblies during sleep and awake immobility [1,2,3]. Disrupting SWRs impairs learning and adaptive behavior, underscoring their central role in hippocampal information processing. Mechanistically, generation of SWRs depends on the interaction of strong CA3 recurrent excitation, fast perisomatic inhibition by parvalbumin (PV)-containing interneurons, and additional synchronizing factors such as gap junctions [4,5]. Despite extensive research, most studies have been conducted in male rodents, and systematic comparisons of SWRs between females and males are lacking.

The hippocampus is functionally and anatomically organized along its longitudinal (dorsoventral) axis. The dorsal (septal/posterior in human) segment of the hippocampus preferentially supports spatial and cognitive aspects of memory, whereas the ventral (temporal, anterior in human) hippocampus is more engaged in affective processing, stress regulation, and anxiety through its strong connectivity with the amygdala, hypothalamus, and medial prefrontal cortex [6,7,8,9,10]. Importantly, several studies indicate that ventral hippocampal manipulations affect anxiety-like behavior in a sex-dependent manner: ventral hippocampal inhibition or lesions modulate anxiety-like responses in male mice, but not necessarily in female mice, and androgen-dependent mechanisms shape excitability in ventral hippocampus–nucleus accumbens circuits [11,12].

Dorsoventral specialization is also evident at the level of network physiology. Both in vivo and in vitro studies demonstrate that SWRs differ between the dorsal and ventral hippocampus in their dynamics and functional coupling [13,14,15,16,17,18,19,20,21]. Dorsal SWRs are associated with spatial memory processing, whereas ventral SWRs preferentially engage limbic circuits and have been implicated in stress-related ensemble reactivation [13,22]. These findings establish dorsoventral differences in SWR dynamics as a key organizing principle of hippocampal activity. Several cellular and synaptic mechanisms that contribute to SWR generation, including inhibitory transmission and excitation–inhibition balance, are known to be modulated by sex-dependent factors such as gonadal steroids [23,24,25,26,27,28,29,30]. These effects provide a basis for expecting sex-related differences in SWR dynamics and for future mechanistic investigations.

Sex differences in behavior and psychopathology linked to ventral hippocampal function are well established. Anxiety disorders, post-traumatic stress disorder, and depression are more prevalent in women, with differences in symptom expression and stress sensitivity [31,32,33]. Experimental studies also demonstrate sex-dependent differences in fear learning and stress-related hippocampal plasticity involving ventral hippocampal–amygdala circuits [34,35]. These findings support the possibility that sex-dependent hippocampal network dynamics contribute to behavioral differences.

SWRs differ along the dorsoventral axis, and several cellular mechanisms underlying these events are subject to sex-dependent modulation. However, direct comparative analyses of SWRs between females and males remain limited, particularly with respect to region-specific differences along the hippocampal longitudinal axis. To address this gap, we systematically compared SWRs and SWR-associated multiunit activity (MUA) in the dorsal and ventral hippocampus of adult female and male rats.

## 2. Materials and Methods

### 2.1. Animals and Hippocampal Slice Preparation

Transverse hippocampal slices (550 μm thick) were obtained from 3–4-month-old female and male Wistar rats housed in the Laboratory of Experimental Animals, Department of Medicine, University of Patras. Animals were maintained under controlled temperature (20–22 °C) and a 12:12 h light–dark cycle with ad libitum access to food and water. All experimental procedures were conducted in accordance with EU Directive 2010/63/EU and were approved by the relevant institutional and regional authorities (reg. number: 5661/37, 18 January 2021). Following deep anesthesia with diethyl ether and decapitation, brains were rapidly removed and placed in ice-cold, oxygenated artificial cerebrospinal fluid (ACSF). Slices were prepared from dorsal and ventral hippocampal regions (0.5–3.5 mm from each pole) and transferred to an interface recording chamber. Slices were continuously perfused with ACSF containing (in mM): 124 NaCl, 4 KCl, 2 CaCl_2_, 2 MgSO_4_, 26 NaHCO_3_, 1.25 NaH_2_PO_4_, and 10 glucose, equilibrated with 95% O_2_ and 5% CO_2_ (pH 7.4), at 30 ± 0.5 °C. Recordings were initiated after at least 90 min of recovery.

### 2.2. Electrophysiology and Signal Processing

Extracellular field potentials were recorded from the CA1 stratum pyramidale using carbon fiber electrodes (7 μm diameter; Kation Scientific, Minneapolis, MN, USA). Signals were amplified (×500), band-pass filtered (0.5 Hz–2 kHz), digitized at 10 kHz (CED 1401-plus, Cambridge Electronic Design, Cambridge, UK), and stored for offline analysis using Spike2 5.03 software (Cambridge Electronic Design, Cambridge, UK). Sharp wave–ripples (SWRs) were identified from downsampled signals (1 kHz) after low-pass filtering (<35 Hz) to isolate the sharp wave component, using threshold-based detection criteria as described below. Events were detected using a threshold-based procedure and confirmed by visual inspection. SWR amplitude was defined as the voltage difference between the peak of the sharp wave and baseline. Event duration was measured at the points where the positive phase intersected baseline. Inter-event interval (IEI) was defined as the time between consecutive SWRs. MUA was extracted from band-pass filtered signals (400–1500 Hz) and detected using a negative threshold crossing criterion, followed by visual verification. MUA was quantified as firing rate (spikes/s).

Spectral analysis of local field potentials was performed using Fast Fourier Transform (FFT). Power spectra were computed from continuous recordings using a Hanning window with a duration of 1.638 s, yielding a frequency resolution of approximately 0.61 Hz. Ripple oscillations were analyzed within the 75–250 Hz frequency range. Ripple peak frequency was defined as the frequency corresponding to the maximum power within this band. Ripple power was quantified as the peak value of the power spectrum within the ripple range. To facilitate visualization of ripple activity and reduce the influence of low-frequency components, spectra are presented within the ripple band (75–250 Hz).

To assess neuronal firing associated with SWRs, peri-event time histograms of MUA were constructed by aligning detected spikes to the peak of each sharp wave event. Histograms were computed using a bin width of 1 ms over a temporal window of ±300 ms around the SWR peak. SWR-associated MUA (MUA-SWR) was defined as the peak firing rate within the peri-event histogram centered on the sharp wave. Baseline MUA (MUA-Base) was estimated from time periods outside the ±100 ms window around the SWR peak, representing activity not directly associated with SWR events. This approach minimizes contamination by SWR-related firing while preserving an estimate of ongoing network activity. Given the relatively low incidence of closely spaced SWRs in the present recordings, contributions from neighboring events were considered negligible.

### 2.3. Statistical Analysis

Statistical analyses were performed using linear mixed-effects models (LMM), with sex and hippocampal region included as fixed factors and animal identity as a random effect to account for repeated measurements. Models were fitted using Restricted Maximum Likelihood (REML). Model assumptions were verified, and the significance of fixed effects and their interaction was assessed using F-tests. Statistical significance was set at *p* < 0.05. The experimental unit in all analyses was the individual rat. Accordingly, statistical analyses were conducted at the animal level, while the number of both rats and slices is reported throughout the text. Data are presented as averages of values obtained from individual rats. Diamond plots display the median, interquartile range (25th and 75th percentiles), mean, 5th and 95th percentiles, and outliers. All analyses were conducted using IBM SPSS Statistics (version 29).

## 3. Results

**Figure 1:**
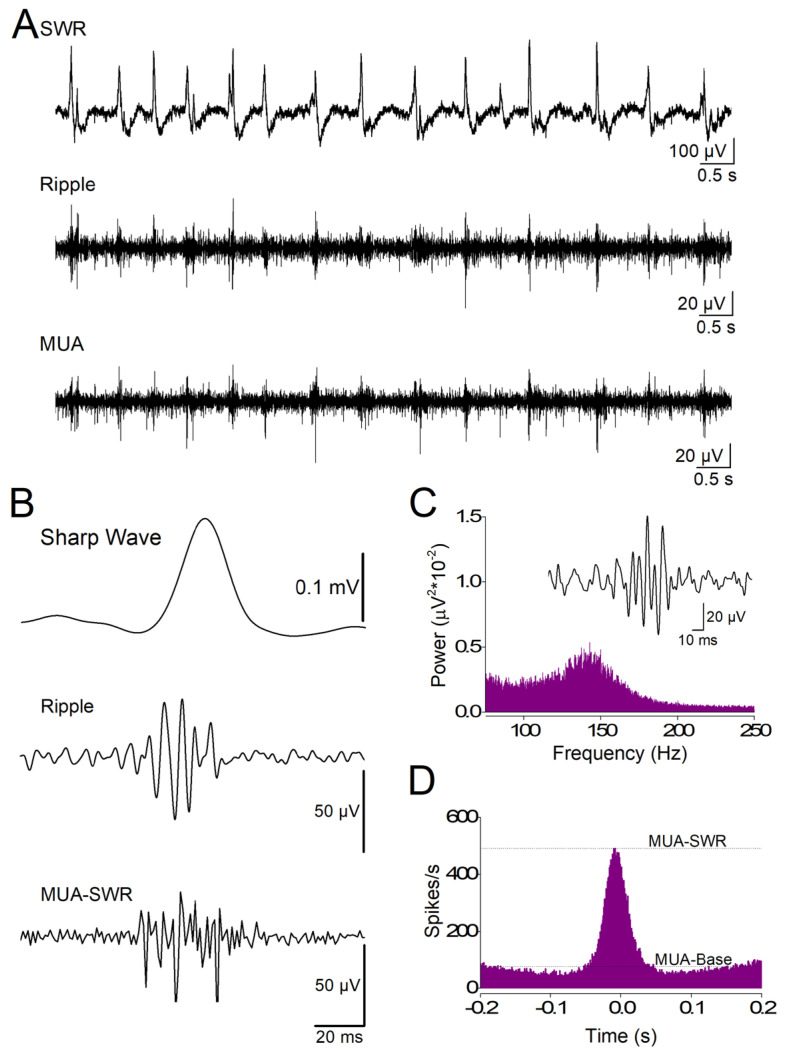
Representative sharp wave–ripples (SWRs) recorded from the stratum pyramidale of the CA1 hippocampal region. (**A**) Raw local field potential (LFP) recording showing multiple SWR events (upper trace). The middle trace shows the same signal band-pass filtered in the ripple range (90–200 Hz), revealing the ripple oscillations. The lower trace shows the signal band-pass filtered (0.4–1.5 kHz), revealing MUA. (**B**) Example of a single SWR event. The raw signal was low-pass filtered (<30 Hz) to isolate the sharp wave component (upper trace), band-pass filtered (100–200 Hz) to reveal the ripple oscillation (middle trace), and high-pass filtered (0.4–1.5 kHz) to extract the associated MUA (bottom trace). (**C**) Power spectrum (Fourier transform) of the LFP signal showing the characteristic ripple-band peak around ~150 Hz. An example of a ripple oscillation is shown in the insert. (**D**) Peri-event time histogram of MUA aligned to the sharp wave peak. Horizontal lines indicate the time windows used to quantify MUA during SWRs (MUA-SWR) and baseline MUA occurring outside SWRs (MUA-Base).

We recorded spontaneously occurring SWRs and MUA from the CA1 stratum pyramidale of the dorsal and ventral hippocampus in female and male rats. From these recordings, we quantified three types of activity: sharp waves, ripple oscillations, and MUA occurring either during SWRs (MUA-SWR) or between SWRs (baseline MUA; Figure 1). Given the known dorsoventral differences in SWR organization [17,18,20,21], analyses were performed to assess the effects of region and sex.

### 3.1. SWR Occurrence Rate

**Figure 2:**
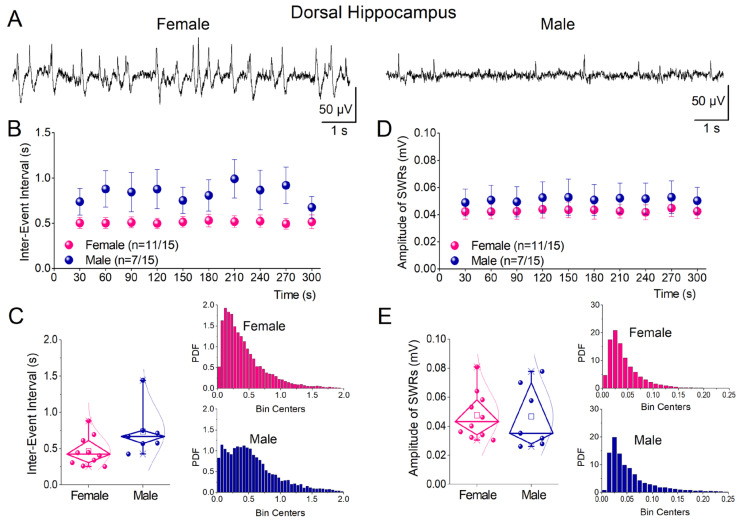
Sex differences in sharp wave–ripple (SWR) activity in the dorsal hippocampus. (**A**) Representative local field potential recordings from dorsal hippocampal slices obtained from a female (left) and a male (right) rat. (**B**) Five-minute time course of inter-event interval (IEI) in the dorsal hippocampus of female (pink) and male (blue) rats. (**C**) Diamond plots (left) and probability density histograms (right) of IEI recorded from the dorsal hippocampus of female and male rats. (**D**) Five-minute time course of SWR amplitude. (**E**) Diamond plots (left) and probability density histograms (right) of SWR amplitude in female and male rats. Data in (**B**,**D**) are shown as mean ± SEM across animals. The number of rats and slices used in these analyses are indicated in the plots (rats/slices). Individual data points in diamond plots represent individual rats, while squares indicate mean values. Histograms are normalized to probability density (PDF) to allow comparison across conditions independent of total event count and include all detected events from a representative 5 s recording segments. Statistical comparisons were performed using linear mixed-effects models (see Table 1).

**Figure 3:**
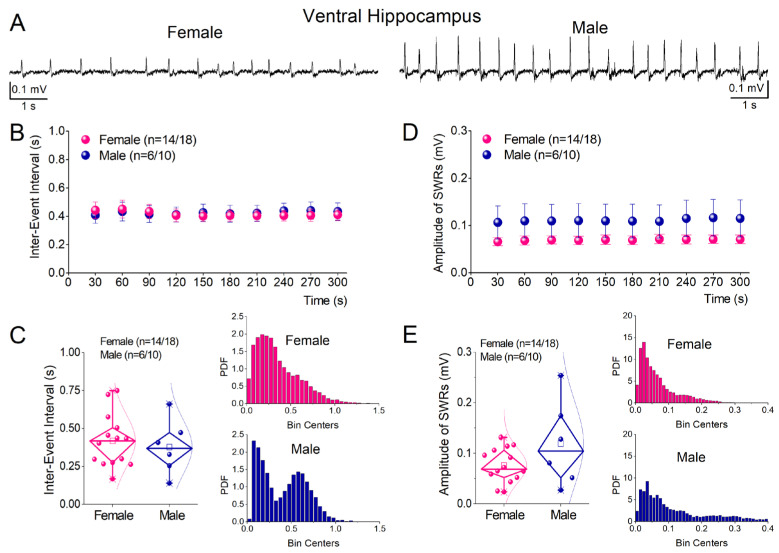
Sex differences in SWR activity in the ventral hippocampus. (**A**) Representative local field potential recordings from ventral hippocampal slices obtained from a female (left) and a male (right) rat. (**B**) Five-minute time course of inter-event interval (IEI) in the ventral hippocampus of female (pink) and male (blue) rats. (**C**) Diamond plots (left) and probability density histograms (right) of IEI recorded from the ventral hippocampus of female and male rats. (**D**) Five-minute time course of SWR amplitude. (**E**) Diamond plots (left) and probability density histograms (right) of SWR amplitude in female and male rats. Data in (**B**,**D**) are shown as mean ± SEM across animals. The number of rats and slices used in these analyses are indicated in the plots (rats/slices). Individual data points in diamond plots represent individual rats, whereas squares indicate mean values. Histograms are normalized to probability density (PDF) to allow comparison across conditions independent of total event count and include all detected events from a representative 5 s recording segment. Statistical comparisons were performed using linear mixed-effects models (see Table 1).

SWR occurrence rate (expressed as the inverse of inter-event interval, IEI) was analyzed using a linear mixed-effects model (Table 1). There was a significant main effect of region (F_(1,26.15)_ = 5.223, *p* = 0.031; dorsal *n* = 18/30, ventral *n* = 20/28; estimated marginal means ± SE: dorsal 0.615 ± 0.074, ventral 0.418 ± 0.043), indicating differences between the dorsal and ventral hippocampus. The main effect of sex did not reach statistical significance (F_(1,26.15)_ = 3.019, *p* = 0.094; female *n* = 18/33, male *n* = 10/25), and the sex × region interaction also did not reach significance (F_(1,26.15)_ = 3.017, *p* = 0.094). Overall, these findings indicate that SWR occurrence rate is significantly modulated by hippocampal region, whereas no statistically significant effect of sex was detected (Figure 2 and Figure 3).

### 3.2. SWR Amplitude

SWR amplitude differed significantly between hippocampal regions (F_(1,23.49)_ = 12.419, *p* = 0.002; dorsal *n* = 18/30, ventral *n* = 20/28; estimated marginal means ± SE: dorsal 0.046 ± 0.035, ventral 0.089 ± 0.036; Table 1), whereas no significant effect of sex was observed (F_(1,23.49)_ = 1.366, *p* = 0.254; female *n* = 18/33, male *n* = 10/25). The interaction between sex and region was not significant (F_(1,23.49)_ = 1.917, *p* = 0.179), indicating that regional differences in SWR amplitude were comparable between males and females (Figure 2 and Figure 3).

### 3.3. Ripple Frequency

Ripple frequency exhibited a significant main effect of region (F_(1,15.45)_ = 13.472, *p* = 0.002; dorsal *n* = 9/10, ventral *n* = 13/14; estimated marginal means ± SE: dorsal 139.16 ± 4.52, ventral 160.82 ± 3.8; Table 1), with no significant effect of sex (F_(1,15.45)_ = 0.622, *p* = 0.442; female *n* = 10/12, male *n* = 9/13) and no interaction (F_(1,15.45)_ = 0.900, *p* = 0.357). These results indicate that ripple frequency is primarily determined by dorsoventral position, independent of sex.

### 3.4. Ripple Power

Ripple power was significantly influenced by both sex and region (Table 1). A significant main effect of sex was observed (F_(1,15.29)_ = 8.883, *p* = 0.009; female *n* = 10/12, male *n* = 8/13), with higher values in females compared to males (estimated marginal means ± SE: female 10.45 ± 2.22, male 3.1 ± 2.27). A significant effect of region was found (F_(1,15.29)_ = 8.270, *p* = 0.011; dorsal *n* = 9/10, ventral *n* = 13/14; estimated marginal means ± SE: dorsal 3.23 ± 1.87, ventral 10.3 ± 2.57). The interaction between sex and region did not reach statistical significance (F_(1,15.29)_ = 3.757, *p* = 0.071).

### 3.5. MUA-Base

**Figure 4:**
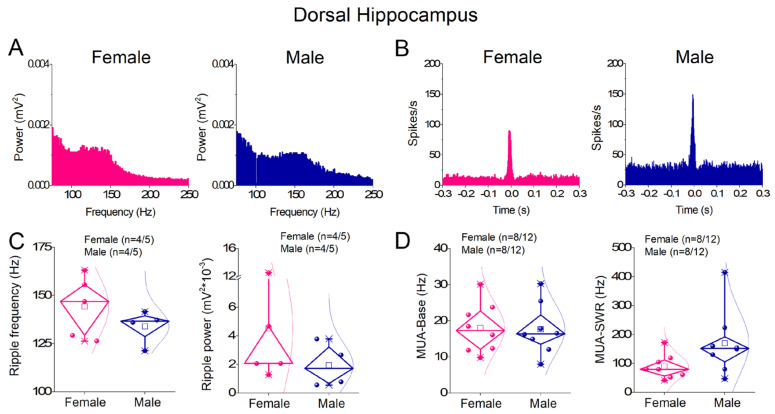
Comparison of ripple oscillations and MUA between female and male dorsal hippocampus. (**A**) Examples of power spectra of local field potentials recorded from dorsal hippocampal slices of a female (left) and a male (right) rat, illustrating ripple-band activity. (**B**) Examples of peri-event time histograms of MUA aligned to the sharp wave peak for female (left) and male (right) rats. (**C**) Diamond plots showing ripple peak frequency (left) and ripple power (right) in the dorsal hippocampus of female (pink) and male (blue) rats. (**D**) Diamond plots summarizing baseline MUA (MUA-Base; left) and SWR-associated MUA (MUA-SWR; right). Data points represent individual rats (circles), while square symbols indicate mean values. The number of rats and slices (rats/slices) is indicated in each panel. Statistical effects of sex, region, and their interaction were assessed using LMM (see Table 1).

**Figure 5:**
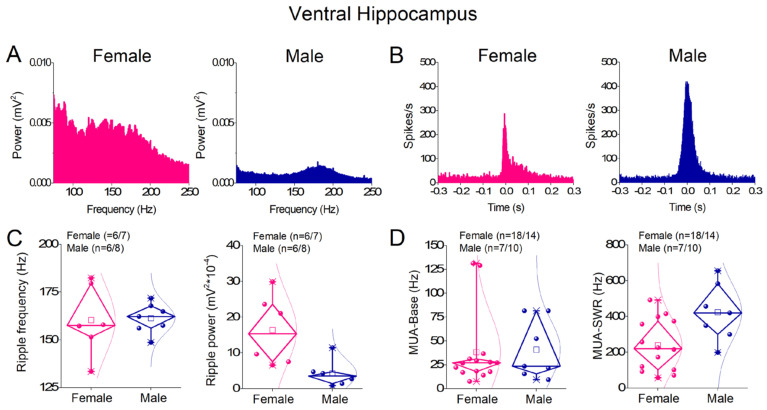
Comparison of ripple oscillations and MUA between female and male ventral hippocampus. (**A**) Examples of power spectra of local field potentials recorded from ventral hippocampal slices of a female (left) and a male (right) rat, illustrating ripple-band activity. (**B**) Examples of peri-event time histograms of MUA aligned to the sharp wave peak for female (left) and male (right) rats. (**C**) Diamond plots showing ripple peak frequency (left) and ripple power (right) in the ventral hippocampus of female (pink) and male (blue) rats. (**D**) Diamond plots summarizing baseline MUA (MUA-Base; left) and SWR-associated MUA (MUA-SWR; right). Data points represent individual rats (circles), whereas squares indicate mean values. The number of rats and slices (rats/slices) is indicated in each panel. Statistical effects of sex, region, and their interaction were assessed using linear mixed-effects models (see Table 1).

MUA-Base showed a significant effect of region (F_(1,20.57)_ = 6.167, *p* = 0.022; dorsal *n* = 16/24, ventral *n* = 25/24; estimated marginal means ± SE: dorsal 17.83 ± 12.44, ventral 39.62 ± 15.02; Table 1), with no significant effect of sex (F_(1,20.57)_ = 0.015, *p* = 0.905; female *n* = 18/26, male *n* = 10/22) and no interaction (F_(1,20.57)_ = 0.026, *p* = 0.873). These findings indicate region-dependent differences in baseline neuronal activity that are not modulated by sex (Figure 4 and Figure 5).

### 3.6. MUA-SWR

In contrast, MUA-SWR exhibited significant effects of both sex and region (Table 1). A strong main effect of sex was observed (F_(1,30.22)_ = 10.645, *p* = 0.003; female *n* = 18/26, male *n* = 10/22), with higher values in males compared to females (estimated marginal means ± SE: male 296.53 ± 70.1, female 163.9 ± 67.17). A significant main effect of region was also found (F_(1,30.22)_ = 24.696, *p* < 0.001; dorsal *n* = 16/24, ventral *n* = 25/24; estimated marginal means ± SE: dorsal 129.22 ± 65.86, ventral 331.2 ± 71.2). The interaction between sex and region was not significant (F_(1,30.22)_ = 1.588, *p* = 0.217, Table 1), indicating that sex differences in SWR-associated neuronal recruitment are consistent across hippocampal regions.

## 4. Discussion

In the present study, we provide a systematic comparison of SWRs and associated MUA between female and male rats along the dorsoventral axis of the hippocampus. Using linear mixed-effects models to account for repeated measurements within animals, we identified robust region-dependent differences, as well as selective effects of sex on specific aspects of SWR-associated activity.

Hippocampal region emerged as a major determinant of multiple SWR properties, consistent with previous reports from our group and others showing marked dorsoventral specialization of hippocampal network dynamics [17,18,20,21]. In contrast, sex effects were more selective. SWR occurrence rate and amplitude did not differ significantly between females and males, and no interaction between sex and region was detected for these measures. These findings indicate that the basic generation and magnitude of SWRs are largely preserved across sexes.

In contrast, sex-dependent differences were observed in measures related to neuronal recruitment and oscillatory structure. In particular, SWR-associated MUA (MUA-SWR) showed a significant main effect of sex, with higher values in males than females, whereas baseline MUA did not differ between sexes. Sex differences were detected at the model level in SWR-associated neuronal recruitment. Because SWR-associated firing depends on coordinated interactions between pyramidal neurons and inhibitory interneurons, these findings point to possible sex-dependent differences in network synchronization mechanisms.

Ripple power was also influenced by sex, with an overall effect indicating differences between females and males, together with a significant effect of region. Although a significant main effect of sex was observed, the absence of a significant interaction between sex and region indicates that conclusions regarding regional specificity are not warranted.

SWR generation depends on coordinated activity within CA3–CA1 circuits and on the balance between excitation and inhibition [1,4]. The absence of sex differences in SWR rate and amplitude suggests that the mechanisms governing event initiation and overall population activation are similar in females and males. In contrast, the higher SWR-associated firing observed in males at the model level suggests differences in how neuronal ensembles are recruited during these events. Such differences may reflect variations in inhibitory network organization or function, including the activity of parvalbumin-positive interneurons that regulate spike timing during ripple oscillations [4,5]. Molecular regulators of inhibitory synapses, such as gephyrin, as well as estrogen receptor–dependent signaling pathways, may contribute to these effects [27,36,37], although direct assessment of these mechanisms was beyond the scope of the present study.

Given that SWRs represent a primary mechanism for hippocampal communication with downstream cortical and subcortical targets [1], differences in SWR-associated neuronal recruitment and ripple structure may influence hippocampal information processing. While SWR rate and amplitude were not influenced by sex, differences in SWR-locked firing could influence the strength or precision of hippocampal output signals.

Several limitations should be considered. First, recordings were performed in acute hippocampal slices, which isolate intrinsic circuit properties but exclude long-range inputs and neuromodulatory influences. Second, estrous cycle stage was not controlled, and potential hormonal influences were not directly examined. Third, several analyses were based on relatively small and/or unbalanced group sizes across conditions (e.g., ripple frequency and ripple power analyses), which may have limited statistical power and reduced sensitivity to detect subtle sex effects or interactions between sex and region. Finally, no a priori power analysis was performed to determine sample size, and no causal manipulations were performed to identify the cellular or synaptic mechanisms underlying the observed differences. Future studies that extend sex- and region-resolved analyses of SWRs to in vivo conditions and disease models with sex-biased prevalence, such as anxiety disorders, depression, schizophrenia, fragile X syndrome, and Alzheimer’s disease, may further clarify the functional relevance of these findings [20,38].

Together, these findings suggest that sex does not substantially alter the generation of hippocampal population events per se but influences the recruitment of neuronal ensembles during these events.

## 5. Conclusions

In conclusion, the present study shows that SWR properties differ robustly along the dorsoventral axis of the hippocampus, and that sex selectively modulates specific aspects of SWR-associated activity. While SWR occurrence rate and amplitude were not significantly influenced by sex, neuronal recruitment during SWRs and ripple oscillatory properties showed sex-dependent differences. These findings provide a descriptive characterization of sex-dependent hippocampal network dynamics and highlight the importance of incorporating sex as a biological variable in studies of hippocampal function.

## Figures and Tables

**Table 1:** Comparisons of sex and hippocampal region effects on SWR-related parameters.

Variable	Effect	F_(df1,df2)_	*p*
Inter-Event Interval (IEI)	Sex	F _(1,26.15)_ = 3.019	0.094
	Region	F _(1,26.15)_ = 5.223	0.031
	Interaction	F _(1,26.15)_ = 3.017	0.094
Amplitude	Sex	F _(1,23.49)_ = 1.366	0.254
	Region	F _(1,23.49)_ = 12.419	0.002
	Interaction	F _(1,23.49)_ = 1.917	0.179
Ripple Frequency	Sex	F _(1,15.45)_ = 0.622	0.442
	Region	F _(1,15.45)_ = 13.472	0.002
	Interaction	F _(1,15.45)_ = 0.900	0.357
Ripple Power	Sex	F _(1,15.29)_ = 8.883	0.009
	Region	F _(1,15.29)_ = 8.270	0.011
	Interaction	F _(1,15.29)_ = 3.757	0.071
MUA-Base	Sex	F _(1,20.57)_ = 0.015	0.905
	Region	F _(1,20.57)_ = 6.167	0.022
	Interaction	F _(1,20.57)_ = 0.026	0.873
MUA-SWR	Sex	F _(1,30.22)_ = 10.645	0.003
	Region	F _(1,30.22)_ = 24.696	<0.001
	Interaction	F _(1,30.22)_ = 1.588	0.217

## Data Availability

All data associated with this study are available from the corresponding author upon reasonable request.

## Abbreviations

The following abbreviations are used in this manuscript:

IEI	inter-event interval
LFP	local field potential
MUA	multiunit activity
MUA-Base	Baseline multiunit activity (outside SWRs)
MUA-SWR	SWR-associated multiunit activity
PV	parvalbumin (interneurons)
SWR	sharp wave-ripple
